# Pharmacology of newly identified nitazene variants reveals structural determinants of affinity, potency, selectivity for mu opioid receptors

**DOI:** 10.1016/j.neuropharm.2025.110512

**Published:** 2025-05-13

**Authors:** Laura B. Kozell, Amy J. Eshleman, Katherine M. Wolfrum, Tracy L. Swanson, Kamryn A. Schutzer, William E. Schutzer, Atheir I. Abbas

**Affiliations:** a VA Portland Health Care System, Portland, OR, 97239, USA; b Department of Psychiatry, Oregon Health & Science University, Portland, OR, 97239, USA; c Department of Behavioral Neuroscience, Oregon Health & Science University, Portland, OR, 97239, USA

**Keywords:** Nitazene, Synthetic opioid, Opioid receptor, Mu opioid receptor, Kappa opioid receptor, Fentanyl, Morphine

## Abstract

Nitazenes, a group of synthetic benzimidazole opioids, are a growing public health threat that have been linked to hundreds of deaths within the last five years. New nitazenes are discovered each year in drug samples and toxicology specimens, necessitating rapid pharmacological characterization. We characterized thirteen nitazenes identified by DEA as chemicals of concern, some that have not been previously characterized. We found that most were very high affinity and potency agonists at the mu opioid receptor (MOR) with very high selectivity for MOR versus other opioid receptors. While bulky benzyl substitutions and a lengthier linker reduced affinity and potency for MOR, the majority of nitazenes tested nonetheless exhibited high-to-very high MOR affinity, potency, and selectivity – often greater than that of fentanyl. Three of the nitazenes exhibited a novel pharmacological pattern, with lower selectivity for MOR versus the kappa opioid receptor (KOR), and with a pharmacological profile that more closely resembles morphine than fentanyl. These findings further delineate the chemical determinants of nitazene pharmacology and identify three of the least MOR-selective nitazenes to date.

## 1. Introduction

The synthesis of a series of ‘nitazene’ benzimidazole derivatives with high potency anti-nociceptive effects was first reported in the 1950s, with several patented and none brought to market(Vandeputte et al., 2021). A large proportion of these nitazene opioids are high affinity, high potency mu opioid receptor (MOR) agonists(Kozell et al., 2024; Vandeputte et al., 2021). More recently, nitazenes have appeared on the illicit/recreational drug market and represent a large and growing proportion of the novel synthetic opioids (NSOs) that are detected by law enforcement and researchers(De Vrieze et al., 2024; Vandeputte et al., 2023). By late 2024, DEA had designated 12 nitazenes as schedule I chemicals(Drug Enforcement Administration, D. C. D., Drug & Chemical Evaluation Section, 2024). Twelve unique nitazenes have been identified in US toxicology specimens and drug samples submitted to one organization since 2019, with N-pyrrolidino etonitazene, metonitazene, and protonitazene being the most common(CFSRE, 2024b).

*In vitro* pharmacological profiling of nitazenes is suggestive of high potential for toxicity even relative to many other opioids, including fentanyl, as most (Kozell et al., 2024; Vandeputte et al., 2021), but not all (Vandeputte et al., 2024), have higher potency and/or affinity at MOR, which is associated with respiratory depression(Miner et al., 2021). In contrast to less selective MOR agonists like morphine, nitazenes tested to date (late 2024) have relatively less activity at the kappa opioid receptor (KOR), which is associated with dysphoria, aversion, and sedation(Dalefield et al., 2022); and the delta opioid receptor (DOR), which is associated with seizures(Pradhan et al., 2011). Nitazenes also show slow dissociation kinetics with reduced sensitivity to naloxone reversal(Alhosan et al., 2024). Consistent with the *in vitro* data, etonitazene, isotonitazene, and n-desethyl isotonitazene are more potent than fentanyl and morphine *in vivo*(Kozell et al., 2024; Vandeputte et al., 2023; Walton et al., 2023). Nitazenes have been repeatedly identified in fatal and non-fatal intoxications around the world(Ameline et al., 2024; Killoran et al., 2024; Montanari et al., 2022; Mueller et al., 2021; Pardi et al., 2023; Schumann et al., 2023). Between January 2020 and December 2021 alone, one laboratory detected nitazenes (predominantly isotonitazene or metonitazene) in blood samples associated with 288 fatal overdoses(Papsun et al., 2022). Eighty-five forensic cases from the US and UK involving five different nitazenes were notable for low-to-sub ng/mL blood concentrations, highlighting the potential for fatal toxicities from very low drug dosages(De Vrieze et al., 2024).

**Fig. 1.**
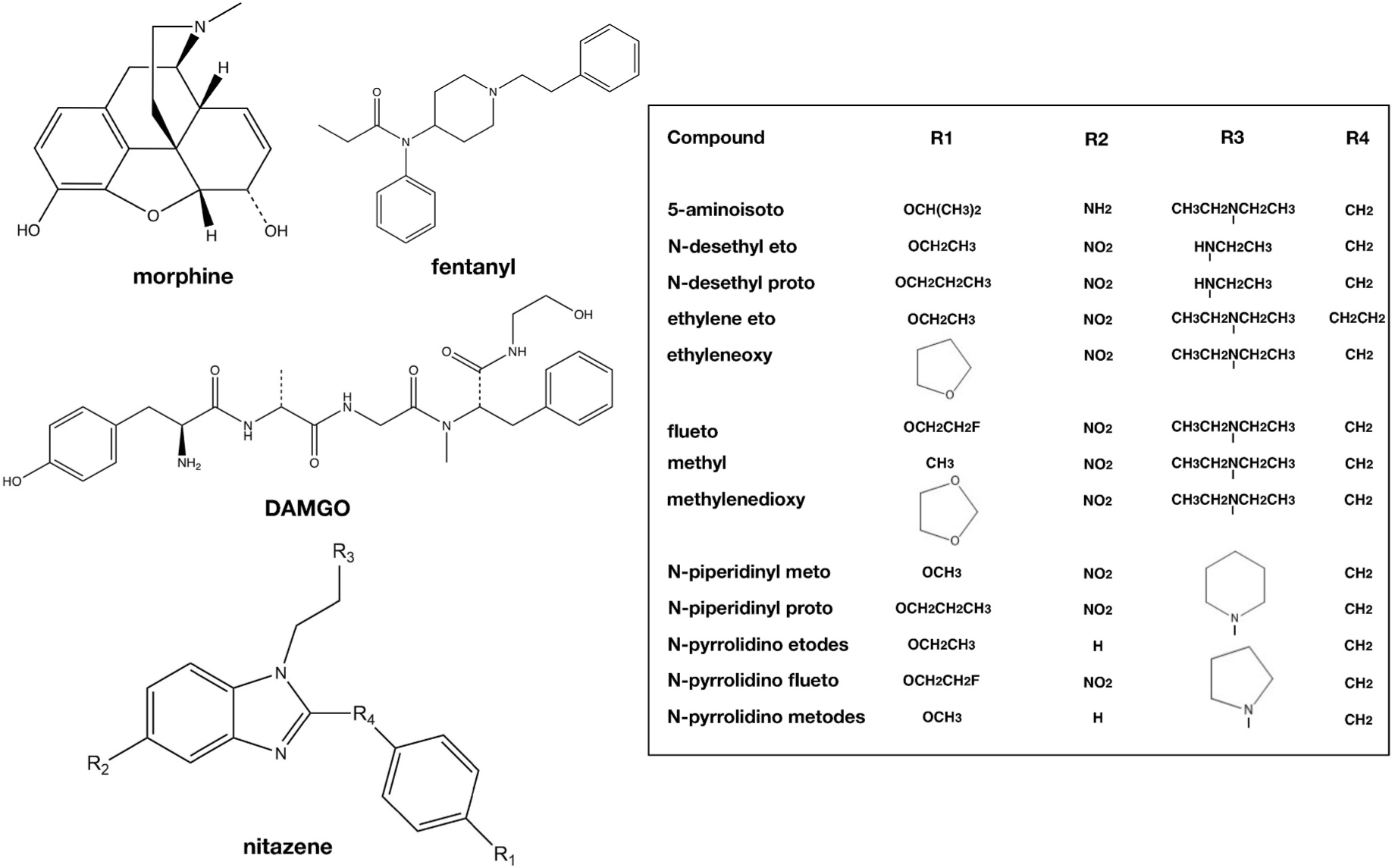
Structures of nitazenes and reference opioid agonists. In the left half of the figure, structures reference mu opioid agonists morphine, fentanyl, and DAMGO are shown. Nitazene base structure is shown with the R1-R4 positions at which substitutions have been made for the drugs tested. The right half of the figure contains a Table that lists all of the tested nitazenes as well as their R1-R4 substitutions. For this figure, “nitazene” has been removed from the compound names for legibility purposes. R3 constituents attach at the nitrogen, signified by a vertical line.

The rapid entry and evolution of nitazenes in the illicit/recreational drug marketplace and their demonstrated toxicity highlight the importance of their rapid pharmacological characterization. Many are identified by authorities and subsequently characterized via pharmacological profiling. More recently, successful efforts have been made to anticipate the evolution of the marketplace by identifying “prophetic” nitazenes, many of which were later identified by law enforcement and/or toxicologists(Vandeputte et al., 2024). In this study, we set out to perform pharmacological profiling of thirteen nitazenes (see Fig. 1 for list and chemical structures) newly identified by DEA as chemicals of concern. We measured affinity, potency, and efficacy at opioid receptors alongside standard opioid compounds for comparison.

## 2. Materials and methods

### 2.1. Chemicals

The radioligands [^3^H]DPDPE (47.4 Ci/mmol), [^3^H]DAMGO (52.2 Ci/mmol), [^3^H]U69,593, (45 Ci/mmol), and [^35^S]GTPγS (1250 Ci/mmol) were purchased from Revvity (Boston, MA). N-desethyl etonitazene, 5-aminoisotonitazene, the hydrochloride salt of N-desethyl protonitazene, and the citrate salts of fluetonitazene, N-pyrrolidino fluetonitazene, ethylene etonitazene, ethyleneoxynitazene, methylnitazene, n-piperidinyl metonitazene, N-piperidinyl protonitazene, N-pyrrolidino etodesnitazene, N-pyrrolidino metodesnitazene, and methylenedioxynitazene (13 nitazenes) were purchased from Cayman Chemical (Ann Arbor, MI). DPDPE ([D-Pen^2^,D-Pen^5^]Enkephalin), DAMGO (Tyr-D-Ala-Gly-N-methyl-Phe-Gly-ol), (±)U50,488H, morphine sulfate pentahydrate, and fentanyl HCl were obtained from the NIDA Drug Supply Program (Bethesda, MD). All other reagents used were obtained from Sigma Scientific (St. Louis, MO).

### 2.2. Cell culture

CHO cells expressing the human receptors were cultured as previously reported(Kozell et al., 2024). CHO-MOR were obtained from Charles River Laboratories (Wilmington, MA, catalog #CT6605), and CHO-KOR and CHO-DOR were received from Stanford Research Institute (SRI, Menlo Park, CA).

### 2.3. Membrane preparation

Cell membranes were prepared as described(Eshleman et al., 2020; Kozell et al., 2024). Cell passage numbers for binding assays were CHO-MOR 3–12; CHO-KOR 10–18; and CHO-DOR 11–20. Cell passage numbers for GTPγS assays were CHO-MOR 7–15; CHO-KOR 12–21; and CHO-DOR 13–19.

### 2.4. Receptor binding

Receptor binding assay conditions were designed to measure receptors in the high affinity state preferred by agonists using sodium-free buffer and as described previously(Eshleman et al., 2020; Kozell et al., 2024). Final volume of the competition binding assay reaction was 1 mL and the concentration of radioligand used was as follows: [^3^H]DAMGO (0.2–1.0 nM), [^3^H]U69,593 (0.2–0.8 nM), [^3^H]DPDPE (0.1–0.8 nM). Duplicate assessments for each test compound were used to determine an averaged single value and for each receptor a minimum of three independent assays were performed. Activation of opioid receptors was tested by measuring the irreversible binding of [^35^S]GTPγS to G protein. Conditions for the assays were optimized for each receptor as previously described(Eshleman et al., 2020; Kozell et al., 2024).

### 2.5. Data analysis

For receptor binding results, nonspecific binding was subtracted, and data were then normalized to specific binding in the absence of drugs. For functional assay results, specific [^35^S]GTPγS binding was calculated by subtraction of basal binding in the absence of drug. GraphPad Prism 10 was used to fit the normalized data via nonlinear regression to generate binding IC50s, functional EC50s and efficacies, and perform statistical comparisons. The Cheng-Prusoff equation(Cheng and Prusoff, 1973) was applied to convert radioligand binding IC_50_ values to Ki values, using the Kd values of 0.273 nM, 0.65 nM, and 0.789 nM for [^3^H] DAMGO at MOR, [^3^H]U69,593 at KOR, and [^3^H]DPDPE at DOR, respectively. % maximum stimulation in functional assays was calculated relative to DAMGO, U50,488H, and DPDPE for MOR, KOR, and DOR, respectively. One-way ANOVA, using −log Kis, −log EC50s, and efficacies of test compounds and standards was used to determine affinity and functional differences. Dunnett’s multiple comparison test was used to statistically assess multiple comparisons with fentanyl. Significance was set at P < 0.05.

## 3. Results

### 3.1. Nitazenes bind to MOR with a wide range of affinities

**Fig. 2.**
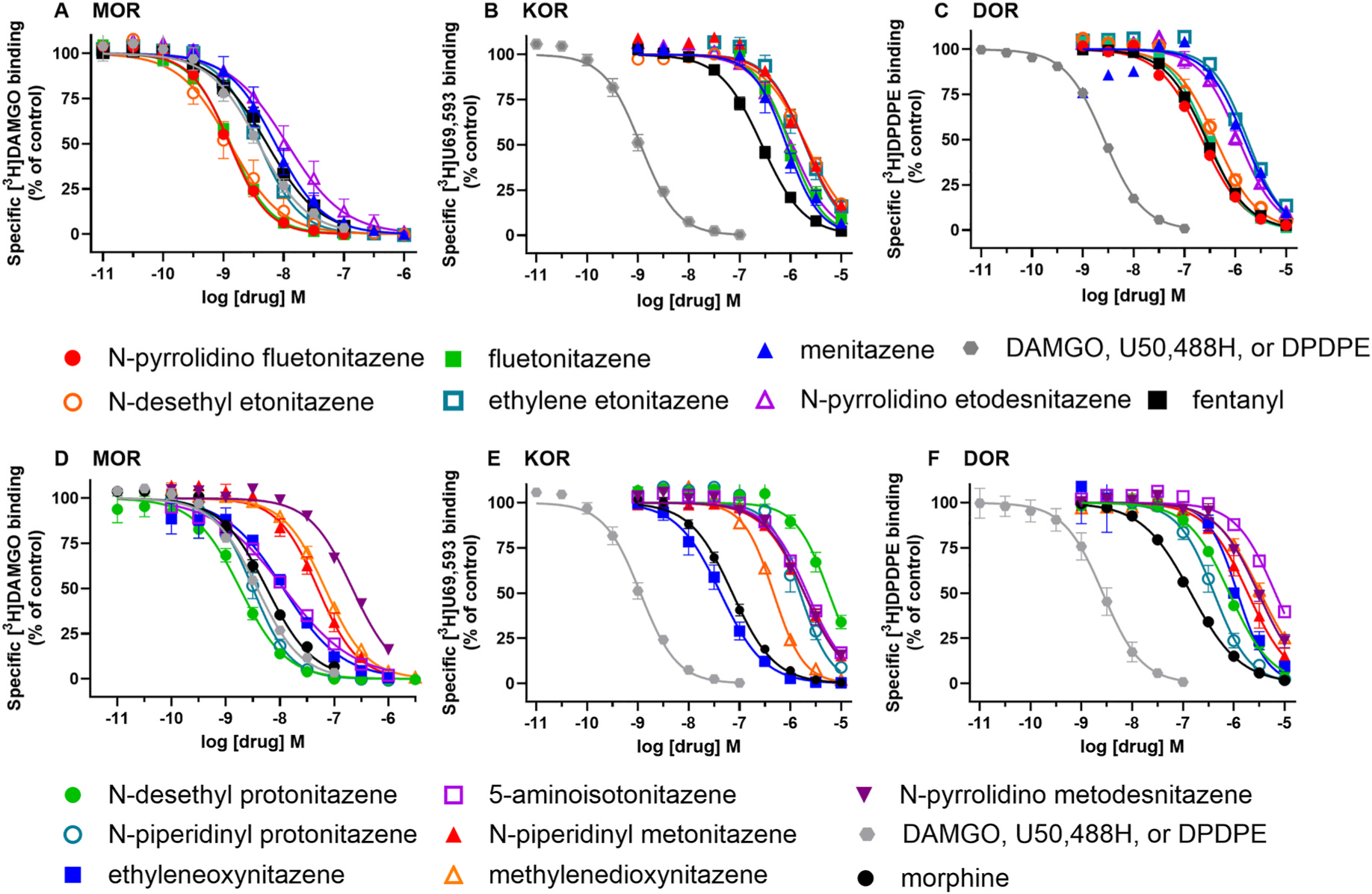
Competition binding curves for substituted nitazenes, fentanyl, morphine, and standards at MOR, KOR, and DOR. The nitazenes have a range of affinities at MOR (A,D), KOR (B,E), and DOR (C,F), though they are relatively right-shifted at KOR and DOR (note x-axis range differences among MOR, KOR, and DOR). For the nitazenes, the mean of 3–7 experiments (MOR), 3–4 experiments (KOR), and 3–4 experiments (DOR) was calculated at each concentration and fit to generate a composite curve for visualization. Selective standards for each receptor are DAMGO (MOR), U50,488H (KOR) and DPDPE (DOR).

**Fig. 3.**
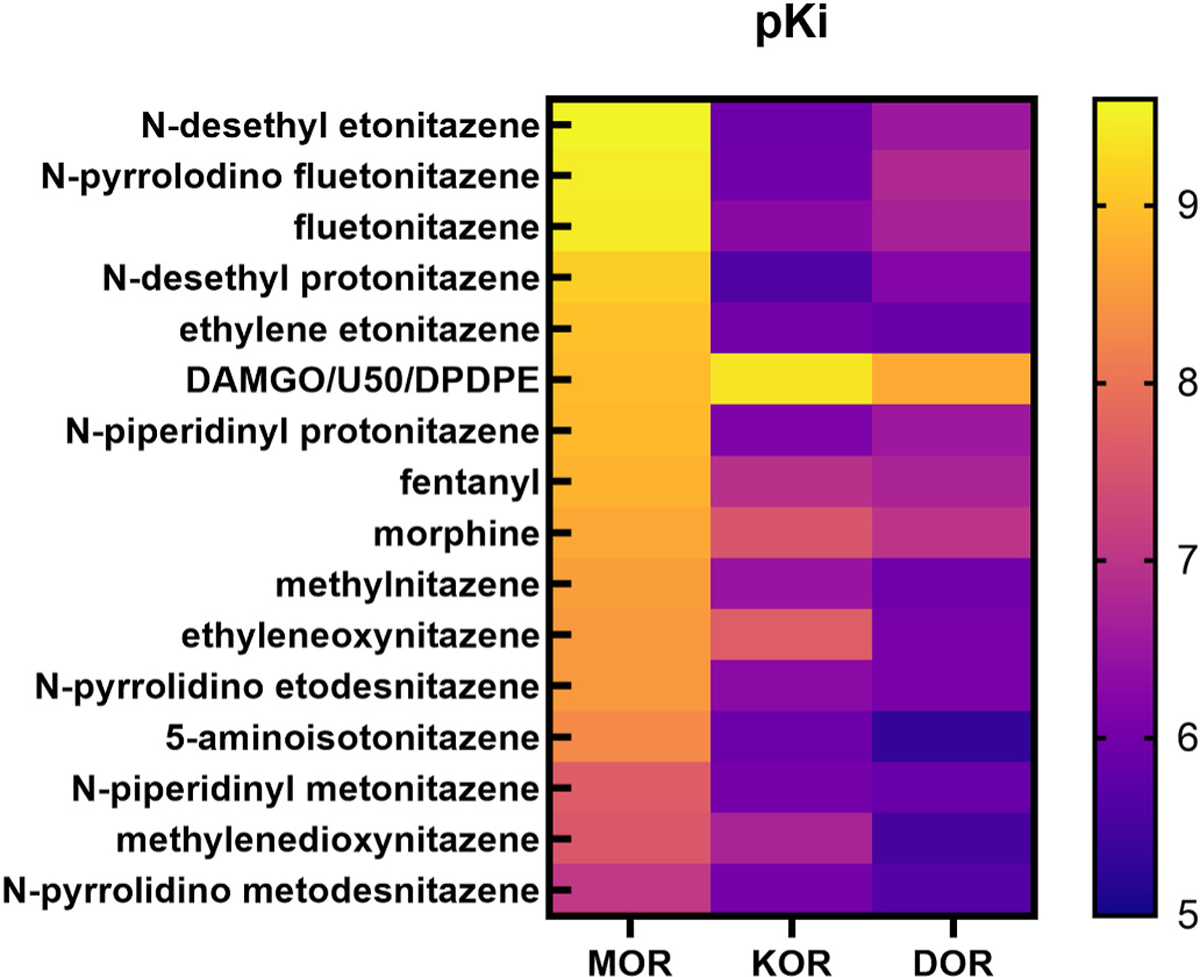
Heat map of substituted nitazenes and standards sorted by pKi values for MOR. Substituted nitazenes and standards were sorted by pKi values for MOR. The higher affinities of most nitazenes for MOR compared to KOR and DOR indicate the selectivity of the compounds for MOR. A handful of nitazenes exhibit a more morphine-like selectivity pattern (for example, see the similarity between the patterns of morphine and ethyleneoxynitazene binding across MOR, KOR, and DOR, in contrast to that of N-desethyl etonitazene).

In MOR agonist [^3^H]DAMGO competition binding assays, the standard agonists fentanyl, morphine, and DAMGO had high affinity for the receptor, with low nanomolar Ki values (Table 1, Figs. 2 and 3; Kis in nM listed in parentheses after each compound). Three compounds, N-desethyl etonitazene (0.317), fluetonitazene (0.354), and N-pyrrolidino fluetonitazene (0.342) had sub-nanomolar Ki values for MOR that were significantly lower than that of fentanyl (1.88) (Table 1, Figs. 2 and 3). N-desethyl protonitazene (0.80) had high sub-nanomolar affinity for MOR that was similar to that of fentanyl. Ethylene etonitazene (1.03), methylnitazene (2.80), ethyleneoxynitazene (3.93), and 5-aminoisotonitazene (4.98) were non-ring-substituted nitazenes that had >1 nM affinities that were also similar to that of fentanyl. Additional compounds with similar affinities to fentanyl included the ring substituted compounds N-piperidinyl protonitazene (1.29) and N-pyrrolidino etodesnitazene (3.36), while the ring-substituted N-piperidinyl metonitazene (24.0) and N-pyrrolidino metodesnitazene (87) had much lower affinity than fentanyl. Methylenedioxynitazene also had lower affinity than fentanyl (28.8).

### 3.2. Nitazenes exhibit lower but measurable affinities for KOR and DOR

In KOR agonist [^3^H]U69,593 competition binding assays, the standard agonist U50,488H had sub-nanomolar affinity for the receptor. Morphine and fentanyl had much lower affinities, with mid-to-high nM Ki values (Table 1, Figs. 2 and 3). The substituted nitazene with the highest affinity for KOR was ethyleneoxynitazene, with a Ki value of 27.6 nM, while the other 12 nitazenes had greater than 100 nM nanomolar to low micromolar affinities. In DOR agonist [^3^H]DPDPE competition binding assays, the standard agonist DPDPE had high affinity with a low nM Ki value. All substituted nitazenes had very low affinities for the DOR, with Ki values ranging from greater than 100 nM to low micromolar values (Table 1, Figs. 2 and 3).

### 3.3. Nitazenes are full agonists at MOR across a wide potency range

**Fig. 4.**
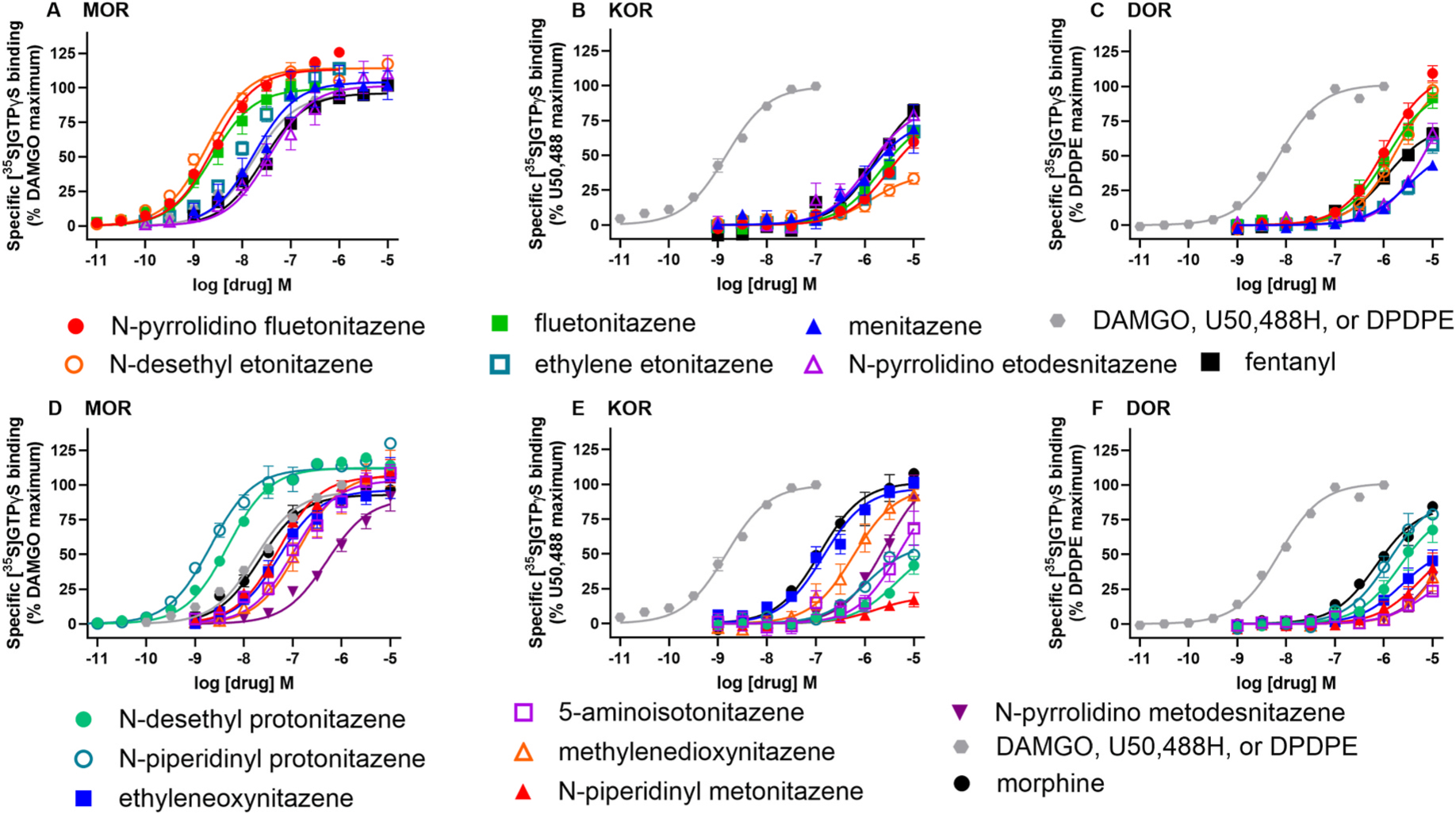
Concentration-response curves of radioligand binding assays of substituted nitazenes, fentanyl, morphine, and standards at MOR, KOR, and DOR. The nitazenes shown have a range of potencies at MOR (A,D), KOR (B,E), and DOR (C,F), though, as with binding, they are relatively right-shifted at KOR and DOR (note x-axis range differences among MOR, KOR, and DOR). At MOR, efficacy is consistently at or near 100 %, whereas at KOR and DOR efficacy varies considerably. For the nitazenes, the mean of 3–5 experiments (MOR), 3–8 experiments (KOR), and 3–4 experiments (DOR) was calculated at each concentration and fit to generate a composite curve for visualization. Specific standards for each receptor are DAMGO (MOR), U50,488H (KOR) and DPDPE (DOR).

**Fig. 5.**
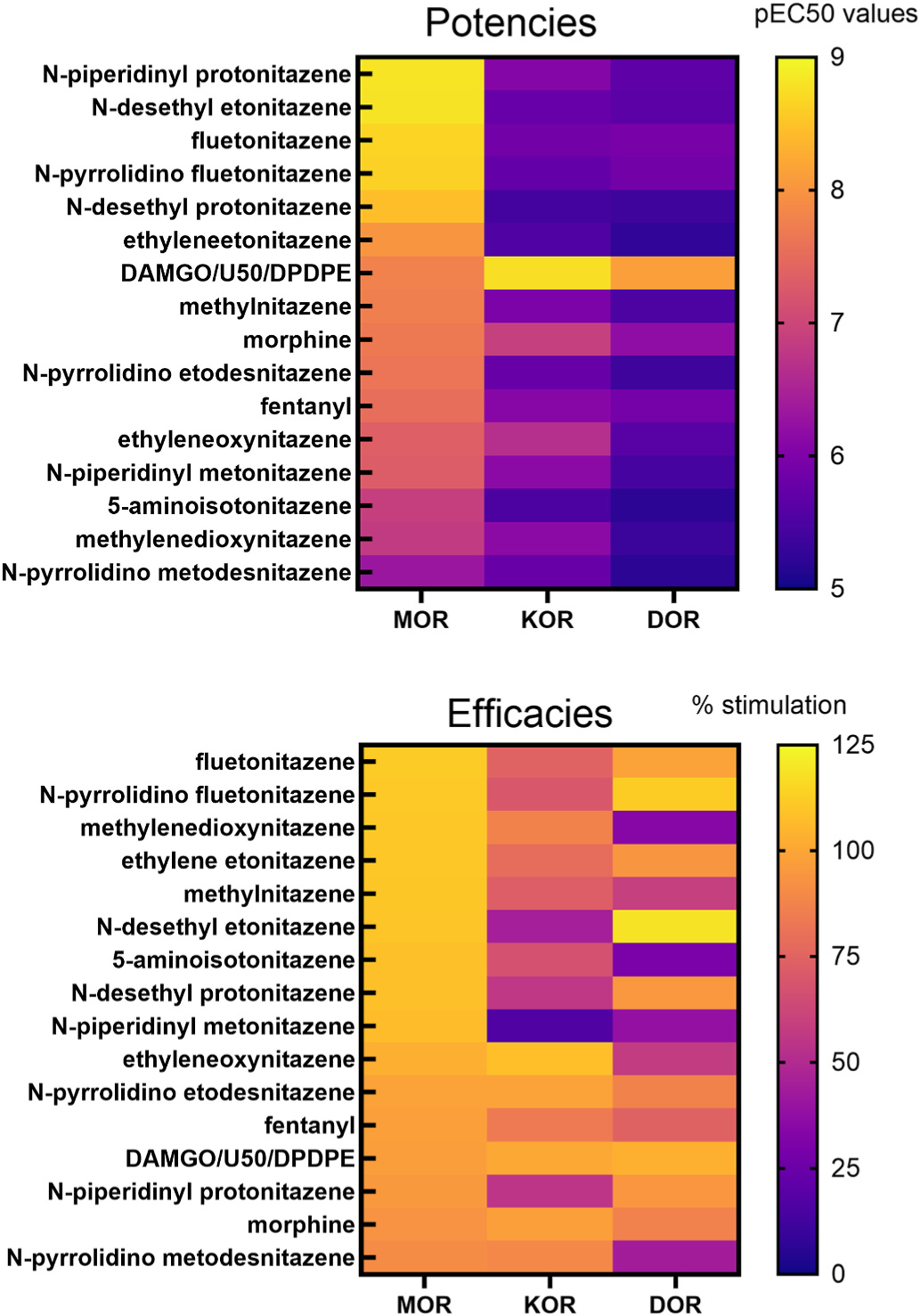
Comparisons of potencies and efficacies in [^35^S]GTPγS binding assays. Potencies (pEC50) of substituted nitazenes and standards at MOR, KOR, and DOR are sorted according to MOR potencies (top panel). Efficacies (% stimulation) are sorted according to MOR efficacies (bottom panel). Note that all compounds were fully efficacious at MOR, but not consistently at DOR and KOR. Some compounds exhibited more balanced potency at MOR and KOR than is typical for nitazenes (i.e., ethyleneoxynitazene versus N-desethyl etonitazene).

**Fig. 6.**
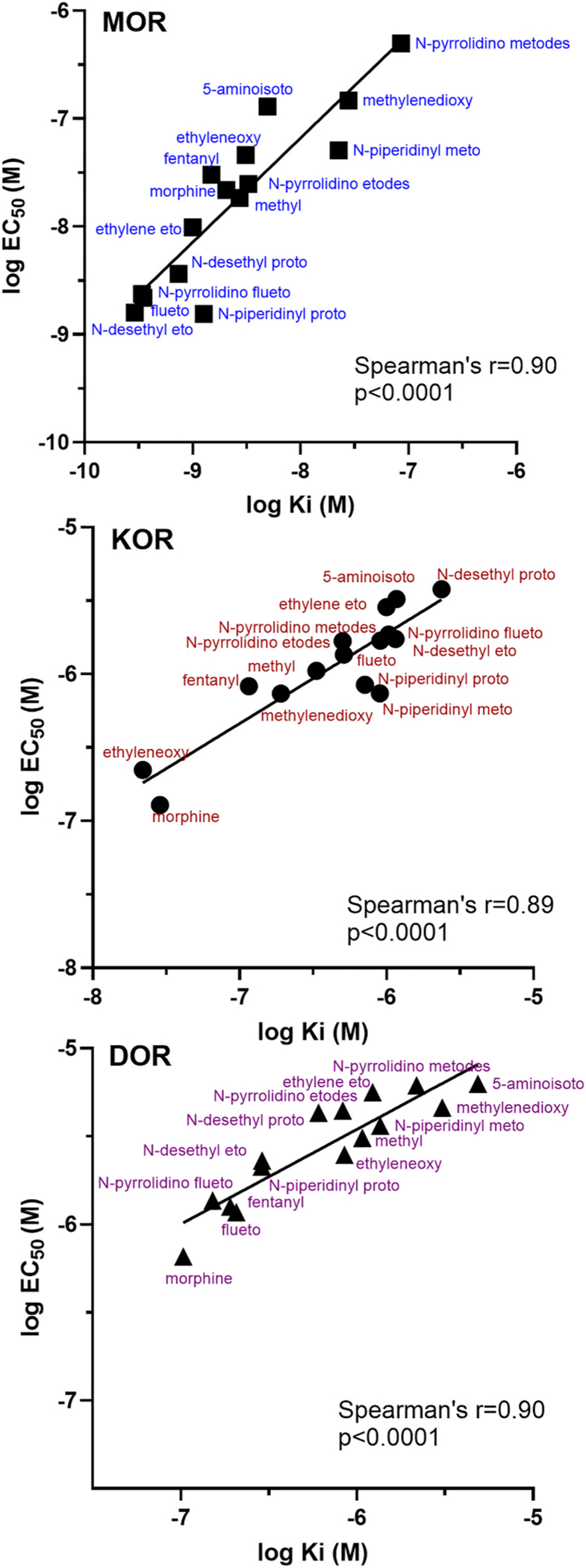
Correlation plots of binding affinities (log Ki values) and functional potencies (log EC50 values) at MOR, KOR, and DOR for substituted nitazenes. Binding affinity and potency are highly correlated (see Spearman’s r and p-values inset). By and large, affinity exceeds potency at MOR, with 5-aminoisotonitazene exhibiting the largest difference, though in some cases affinity and potency values are very similar (N-piperidinyl protonitazene). For this figure, “nitazene” has been removed from the names for legibility purposes and the x-axis and y-axis within a subgraph have the same ranges.

In MOR agonist [^35^S]GTPγS functional assays, all compounds tested were full agonists with efficacies similar to fentanyl, although potencies differed between the compounds (Table 2 and Figs. 4 and 5). The standard agonists fentanyl, morphine, and DAMGO had EC50 values ranging from 17.8 to 30.4 nM, with DAMGO having significantly higher potency than fentanyl. Six compounds, N-piperidinyl protonitazene (1.57), N-desethyl etonitazene (1.60), fluetonitazene (2.26), N-pyrrolidino fluetonitazene (2.40), N-desethyl protonitazene (3.78), and ethylene etonitazene (10.3) had low nanomolar potencies for MOR activation and were significantly more potent than fentanyl. Four compounds, methylnitazene (20.1), N-pyrrolidino etodesnitazene (27.1), ethyleneoxynitazene (48.0), and N-piperidinyl metonitazene (53) had similar potencies as fentanyl. Compounds with lower potencies compared to fentanyl included methylenedixoynitazene (154), 5-aminoisotonitazene (144), and N-pyrrolidino metodesnitazene (498). There was a significant correlation between log Ki and log EC50 values for MOR (Spearman’s r = 0.90, p value < 0.001, Fig. 6).

### 3.4. Nitazenes are partial to full agonists that exhibit lower but measurable potencies at KOR and DOR

In KOR functional assays, U50,488H, and morphine had significantly higher potencies than fentanyl (Table 2 and Figs. 4 and 5). The potencies of all the substituted nitazenes, which were high nanomolar to low micromolar, did not differ from that of fentanyl (p > 0.05). Two compounds had maximal effects that differed significantly from fentanyl: N-desethyl etonitazene and N-piperidinyl metonitazene had lower efficacies. There was a significant correlation between log Ki and log EC50 values for KOR (Spearman’s r = 0.89, p value < 0.001, Fig. 6). Note that the correlation is still significant when morphine and ethyleneoxynitazene are excluded (Spearman’s r = 0.84, p value = 0.0005), indicating that the correlation is not driven only by these higher affinity and potency compounds. In DOR functional assays, fentanyl had low micromolar potency and only DPDPE had higher potency. Morphine, N-desethyl etonitazene, ethyleneoxynitazene, fluetonitazene, N-piperidinyl protonitazene, and N-pyrrolidino fluetonitazene had low micromolar potencies similar to fentanyl. The rest of the substituted nitazenes, 5-aminoisotonitazene, N-desethyl protonitazene, ethylene etonitazene, methylnitazene, methylenedioxynitazene, N-piperidinyl metonitazene, N-pyrrolidino etonitazene, and N-pyrrolidinyl metonitazene had lower potency with higher micromolar EC50 values. Fentanyl was a partial-to-full agonist at DOR (74.0 % stimulation). DPDPE, N-desethyl etonitazene, fluetonitazene, and N-pyrrolidino fluetonitazene had higher efficacies, while 5-aminoisotonitazene, methylenedioxynitazene, N-piperidinyl metonitazene, and N-pyrrolidinyl metonitazene had lower efficacies than fentanyl. There was a significant correlation between log Ki and log EC50 values for DOR (Spearman’s r = 0.90, p value < 0.001, Fig. 6).

### 3.5. Selectivity of tested nitazenes for MOR versus KOR and DOR

**Fig. 7.**
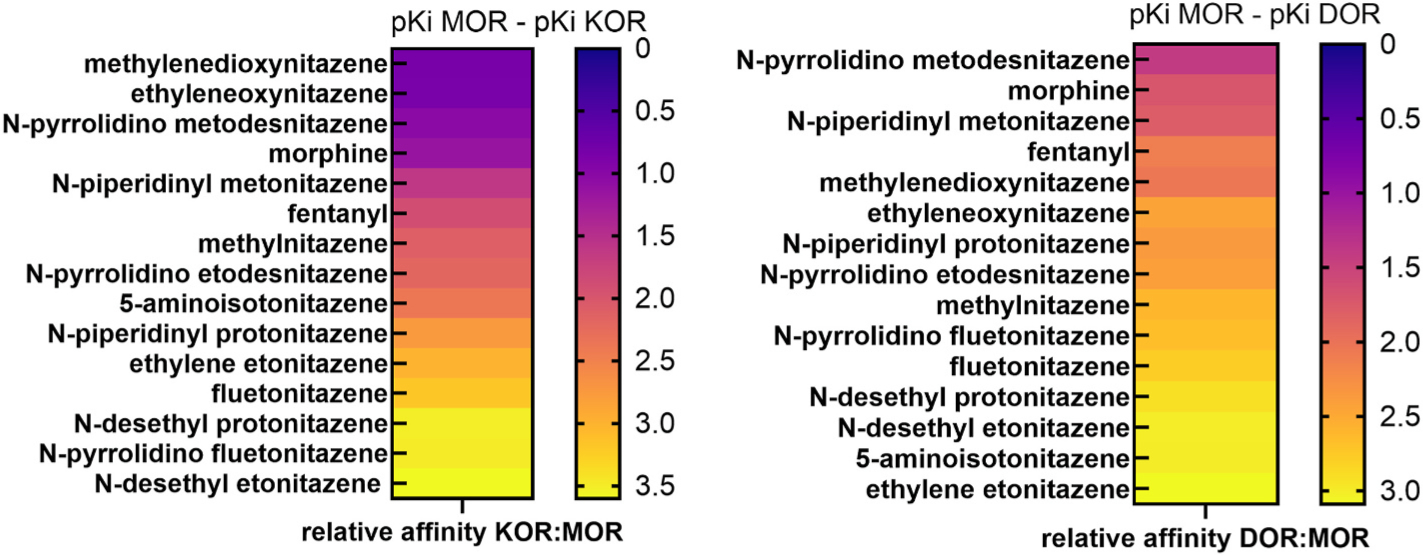
Comparisons of MOR versus KOR and MOR versus DOR selectivity. To examine selectivity for MOR versus KOR and DOR, we calculated and plotted heat maps of the difference between MOR and KOR pKis (MOR vs KOR selectivity) and MOR and DOR pKis (MOR vs DOR selectivity). Zero equates to no selectivity, 0.5 to a half-log lower Ki for MOR versus KOR or DOR, 1.0 to a full-log lower Ki (i.e., a 10-fold difference in affinity), and so on. Some compounds exhibited more balanced affinity at MOR and KOR than is typical for nitazenes (i.e., methylenedioxynitazene, ethyleneoxynitazene, and N-pyrrolidino metodesnitazene versus N-desethyl etonitazene), overall exhibiting a more morphine-like pharmacological profile than previously described nitazenes.

While most of the nitazenes had high selectivity for MOR versus KOR, some of the nitazenes tested appeared to exhibit lower selectivity for MOR versus KOR (5–10-fold). This is atypical compared to previously reported nitazenes that are typically >100-fold selective for MOR. We plotted the difference between the MOR pKi and KOR pKi as well as the difference between the MOR pKi and the DOR pKi for each chemical tested, sorting the results from low to high and plotting as a heat map (Fig. 7). We found that three of the nitazenes tested – ethyleneoxynitazene, methylenedioxynitazene, and N-pyrrolidino metodesnitazene – indeed exhibited lower selectivity for MOR versus KOR while retaining high selectivity for MOR versus DOR. Overall, this pharmacological pattern at opioid receptors suggests that these nitazenes are the most morphine-like nitazenes described to date and highlight previously undescribed structural variation which can decrease the selectivity of nitazenes for MOR versus KOR.

## 4. Discussion

As we(Kozell et al., 2024) and others(Glatfelter et al., 2023; Vandeputte et al., 2022, 2023, 2024) have previously noted, nitazenes as a group tend to be full agonists and have very high affinity, potency, and selectivity (relative to other opioid receptors) for MOR, in some cases exceeding that of fentanyl many-fold. In a group of 19 nitazenes we described last year, only two had a Ki for MOR above 10 nM and 12 out of 19 tested were below 1 nM(Kozell et al., 2024). Furthermore, all were highly selective for MOR – 46-fold–2580-fold for MOR relative to KOR and >170-fold for MOR relative to DOR. Potencies were more variable, but all except five exceeded that of fentanyl. In the group of 13 nitazenes tested here, approximately half were higher or comparable affinity and potency relative to fentanyl. Furthermore, while most were highly selective, three exhibited < 10-fold selectivity for MOR versus KOR.

Notably, many of the nitazenes tested here are just beginning to be characterized with respect to their basic pharmacology, and three (fluetonitazene, N-pyrrolidino fluetonitazene, and methylenedioxynitazene) are, to our knowledge, uncharacterized pharmacologically (Vandeputte and Stove, 2025). N-desethyl etonitazene was recently detected in a drug checking sample and subsequently noted to exhibit < 5 nM potency using two non-cAMP readouts at the MOR(Monti et al., 2024). N-desethyl protonitazene has been reported to have a potency of about 0.6 nM using a cAMP readout and close to 4 nM with an arrestin-based readout (De Vrieze et al., 2024). It has also been reported as a metabolite of protonitazene as demonstrated using liver microsomes and human hepatocytes(Ameline et al., 2024; Kanamori et al., 2024). We find that N-desethyl protonitazene has sub-nanomolar affinity and high potency (< 5 nM) for MOR. Our estimate is comparable though slightly higher than the previously reported estimate cited above. Assay differences are a likely factor given the differences in fentanyl potency between the two studies (~30 nM in this study versus ~2.2 nM in the above study). Nonetheless, these values suggest that protonitazene has at least one metabolite that is highly active. Of note, while N-desethyl protonitazene and N-desethyl etonitazene have comparable MOR affinity to N-desethyl isotonitazene, which we previously estimated to be approximately 0.25 nM(Kozell et al., 2024), they are 50–100-fold less potent in the functional assay than N-desethyl isotonitazene. This suggests that the difference at R1 (see Fig. 1) has limited impact on binding but dramatically affects agonist activation properties. Further reinforcing the importance of R1 size/length, we found that methylnitazene has high selectivity for MOR but lower affinity and potency than many other nitazenes, comparable but slightly lower than that of fentanyl. Here, our estimates for Ki and EC50 are somewhat discrepant compared to a recent report (~2 nM versus 57 nM Ki and ~20 nM versus 70 nM EC50), even relative to fentanyl (affinity and potency were comparable in our study whereas they were previously reported to exhibit lower affinity and potency compared to fentanyl)(Vandeputte et al., 2024). Fluetonitazene, which was reported in 2024 in an EU Early Warning System notification(EMCDDA, 2024), retains the very high affinity, potency, and selectivity of etonitazene to which it is closely related. Overall, our findings reinforce previous observations that alkoxy chain lengths of 2- to 3- are optimal with respect to maximizing affinity and potency at MOR, with the least bulky substitutions being associated with the lowest MOR affinity and potency(Glatfelter et al., 2023; Kozell et al., 2024), and our findings of relatively lower MOR affinity and potency agree with a recent report(Vandeputte et al., 2024).

Ethyleneoxynitazene is a cyclized analogue of etonitazene that was first identified in Europe in February 2023 and reported recently to have moderate affinity and potency at MOR comparable but lower than fentanyl(Vandeputte et al., 2024). We similarly find that it has lower affinity and potency than fentanyl, though our single digit estimates differ by 5–10-fold relative to the previous report, likely because our assays were performed using overexpressed human MOR whereas the previous estimate used homogenized rat brain tissue. Interestingly, we find that ethyleneoxynitazene is among the least selective nitazenes reported to date, with only approximately 5–6-fold selectivity for binding to and activating MOR relative to KOR. It retains approximately 100-fold selectivity for MOR relative to DOR. Methylenedioxynitazene was reported in August 2024 in a drug monograph by the Center for Forensic Science and Education (CFSRE) after detection in two UK toxicology samples and in drug material from Missouri(CFSRE, 2024a). Other than a very recent investigation of its metabolism in human liver microsomes, it remains largely uncharacterized(Huang et al., 2024). We find that its affinity, potency, and selectivity for MOR versus KOR are well below that of most nitazenes, including most tested in this study, and below that of fentanyl. It is also has a higher Ki and EC50 and lower selectivity for MOR than ethyleneoxynitazene, which has a less bulky R1 substitution, again reinforcing the importance of an intermediate size/length of that substitution.

Ethylene etonitazene was first reported decades ago to be a highly potent antinociceptive and identified in early 2024 in relation to illicit/recreational use(Vandeputte et al., 2024). Consistent with a recent report(Vandeputte et al., 2024), we find that lengthening of the R4 linker region lowers the affinity and potency relative to etonitazene, though it remains higher affinity and potency than fentanyl and is highly selective (>1000-fold) for MOR relative to KOR and DOR. Similar to our previous report(Kozell et al., 2024) and another recent report(De Vrieze et al., 2024), we found that piperidinyl or pyrrolidino substitutions at R3 have limited impact on potency, with R1 and R2 substitutions being of considerably greater importance. Our measurements of affinity are consistent with that view. Overall, this study provides a substantial pharmacological characterization of the thirteen nitazenes in this report at opioid receptors, some of which were previously uncharacterized or lightly characterized, especially at KOR and DOR. In some cases, noted earlier in the discussion, the nitazenes we describe have not been examined previously with respect to their opioid receptor pharmacology. Where data exists, our findings are largely consistent with previous reports, with methylnitazene being the most discrepant. One important limitation of this study is related to the KOR and DOR functional data. Due to the low potency of a large subset of the chemicals (the top plateau is not well delineated for most of the chemicals at the 10 μM concentration), the curves are to varying extents extrapolated. With a few potential exceptions, this is unlikely to have a large effect on the potency determination as most of the chemicals reach or almost reach 50%. However, the extrapolation does mean that the efficacy determinations for KOR and DOR can reasonably be considered more uncertain. For binding studies and selectivity estimates (which are based on the binding estimates), there is less of an extrapolation concern for a few reasons. First, we have fuller coverage of the binding curves; second, extrapolating to no specific binding at high concentrations is a reasonable assumption for orthosteric ligands; and third, the Hill slopes are highly consistent among the nitazenes.

To conclude, we wish to emphasize a few critical points. First, the illicit/recreational marketplace for MOR agonists is evolving rapidly. New nitazenes are detected frequently and reported by toxicologists and/or early warning systems. Given the rapid evolution of the illicit/recreational nitazene market and their well-established physiologic toxicity, identification by toxicologists followed by rapid pharmacological characterization is critical. An even more proactive approach, in which several ‘prophetic’ nitazenes were rationally identified prior to their detection in the illicit/recreational marketplace, has already met with success in that it anticipated their arrival as demonstrated by toxicological testing(Vandeputte et al., 2024). Second, three of the nitazenes tested – ethyleneoxynitazene, methylenedioxynitazene, and N-pyrrolidino metodesnitazene – exhibit low selectivity for MOR versus KOR (5–10-fold). This is atypical compared to most previously reported nitazenes that are typically >100-fold selective for MOR. Ethyleneoxynitazene and methylenedioxynitazene have a little studied substitution at R1, suggesting that ring substitutions at R1 may reduce selectivity for MOR. N-pyrrolidino metodesnitazene is surprising in that metodesnitazene and previous N-pyrrolidino nitazenes have all been highly selective for MOR. The reason for its relative lack of selectivity is unclear. Overall, the pattern of affinity, potency, and selectivity make ethyleneoxynitazene, methylenedioxynitazene, and N-pyrrolidino metodesnitazene the first nitazenes that appear more ‘morphine-like’ in their pharmacology and present the possibility that some nitazene variants could even be KOR > MOR agonists. A third point is that the nitazenes we tested here exhibit overall lower affinity and potency relative to many previously better characterized nitazenes. For example, in our previous examination of nineteen nitazenes, 13/19 had sub-nanomolar affinity and 4/13 sub-nanomolar potency for MOR (Kozell et al., 2024), whereas in this current group, only 4/13 exhibit sub-nanomolar MOR affinity and none sub-nanomolar potency. Finally, non-opioid activities of nitazenes are largely uncharacterized even though off-target action appears to be a critical mediator of some opioid toxicities(Torralva et al., 2020). Off-target characterization presents an important future area of investigation for nitazenes that demonstrate real world toxicity.

## 5. Conclusions

Nitazenes represent a growing public health threat and have been linked to non-fatal toxicity and hundreds of fatalities. We pharmacologically characterized thirteen emerging nitazenes at MOR, KOR, and DOR. We found that many have very high affinity and potency, are highly selective for MOR, and all are full agonists at MOR – typical of many previously characterized nitazenes. Our findings highlight key structure activity relationships, including newly described motifs that reduce selectivity for MOR versus KOR, conferring a more morphine-like pattern of binding and function at opioid receptors. Overall, these findings support continued rapid pharmacological characterization of emerging nitazenes.

## Figures and Tables

**Table 1:** MOR, KOR and DOR Binding Ki values for substituted nitazenes.

Drug	MOR [^3^H] DAMGO Ki (nM) ± SEM (n)	KOR [^3^H] U69,593 Ki (nM) ± SEM (n)	DOR [^3^H] DPDPE Ki (nM) ± SEM (n)

fentanyl	1.88 ± 0.23 (18)	134 ± 20 (16)	196 ± 13 (12)
morphine	2.18 ± 0.20 (16)	30.5 ± 2.7 (18)****	106.4 ± 7.9 (12)****
DAMGO	1.26 ± 0.13 (18)		
U50,488H		0.519 ± 0.071 (21)****	
DPDPE			1.94 ± 0.12 (14)****
5-aminoisotonitazene	4.98 ± 0.30 (4)**	1179 ± 85 (4)****	4940 ± 500 (4)****
N-desethyl etonitazene	0.317 ± 0.064 (4)****	1154 ± 76 (3)****	299 ± 55 (3)
N-desethyl protonitazene	0.80 ± 0.13 (5)	2400 ± 320 (3)****	613 ± 49 (5)
ethylene etonitazene	1.03 ± 0.10 (4)	1080 ± 270 (3)****	1229 ± 44 (3)****
ethyleneoxynitazene	3.93 ± 0.96 (5)	27.6 ± 9.2 (5)****	860 ± 89 (3)****
fluetonitazene	0.354 ± 0.013 (3)***	540 ± 110 (3)***	207 ± 10 (3)
methylnitazene	2.80 ± 0.52 (3)	345 ± 48 (4)**	1090 ± 110 (3)****
methylenedioxynitazene	28.8 ± 3.1 (6)****	192 ± 14 (3)	3450 ± 890 (6)****
N-piperidinyl metonitazene	24.0 ± 4.2 (4)****	930 ± 160 (3)****	1440 ± 360 (3)****
N-piperidinyl protonitazene	1.29 ± 0.15 (4)	770 ± 190 (4)****	300 ± 63 (3)
N-pyrrolidino etodesnitazene	3.36 ± 0.44 (4)	503 ± 27 (3)****	850 ± 110 (3)****
N-pyrrolidino fluetonitazene	0.342 ± 0.014 (3)***	1110 ± 260 (3)****	153 ± 12 (3)
N-pyrrolidino metodesnitazene	87 ± 10 (4)****	940 ± 170 (3)****	2210 ± 260 (3)****

(n) is the number of biological replicates, each of which is conducted in duplicate. The Hill slopes ranged between −0.32 and −1.51 for MOR, −0.81 and −2.03 for KOR, and −0.74 and −2.24 for DOR.

* p < 0.05

** p < 0.01

*** p < 0.001

**** p < 0.0001.

We performed one-way ANOVA followed by Dunnett’s multiple comparison test to compare the log Ki values of each compound to the log Ki values of fentanyl at each receptor.

**Table 2:** MOR, KOR, and DOR functional values for substituted nitazenes.

Drug	MOR EC50 (nM) ± SEM % max stimulation (n)	KOR EC50 (nM) ± SEM % max stimulation (n)	DOR EC50 (nM) ± SEM % max stimulation (n)

fentanyl	30.4 ± 1.2	970 ± 220	1390 ± 270
	97.4 ± 2.2 % (6)	83.8 ± 6.4 % (7)	74.0 ± 3.7 % (7)
morphine	22.3 ± 2.2	134 ± 18**	673 ± 70
	93.1 ± 2.4 % (6)	97.1 ± 3.9 % (6)	86.7 ± 2.3 % (7)
DAMGO	17.8 ± 1.4*		
	97.0 ± 1.4 % (12)		
U50,488H		2.04 ± 0.39****	
		100.33 ± 0.60 % (11)	
DPDPE			8.4 ± 1.0****
			103.1 ± 1.1 %**** (13)
5-aminoisotonitazene	144 ± 35****	3450 ± 640	6700 ± 1100****
	108.3 ± 4.6 % (4)	67 ± 16 % (4)	31.0 ± 6.3 %**** (5)
N-desethyl etonitazene	1.60 ± 0.10****	3040 ± 900	2450 ± 570
	109.6 ± 5.7 % (4)	44.7 ± 3.7 %*** (6)	118.80 ± 0.72 %**** (3)
N-desethyl protonitazene	3.78 ± 0.63****	4800 ± 1700	4400 ± 570***
	108.1 ± 3.5 % (4)	55.8 ± 9.9 % (4)	94.8 ± 8.0 % (4)
ethylene etonitazene	10.3 ± 1.0***	3150 ± 360	5800 ± 810****
	110.4 ± 2.4 % (3)	78.1 ± 2.3 % (4)	87 ± 13 % (4)
ethyleneoxynitazene	48.0 ± 7.1	255 ± 71	2670 ± 750
	103 ± 11 (4)	108.0 ± 4.6 % (5)	57.7 ± 8.0 % (3)
fluetonitazene	2.26 ± 0.43****	1680 ± 460	1260 ± 270
	111.4 ± 8.1 % (3)	74.1 ± 6.0 % (4)	98.1 ± 5.9 %* (4)
methylnitazene	20.1 ± 5.1	1056 ± 65	3190 ± 540*
	110 ± 0 % (3)	72 ± 15 % (3)	59.2 ± 5.0 % (3)
methylenedioxynitazene	154 ± 32****	780 ± 180	5400 ± 1600***
	110.7 ± 9.1 % (3)	86.9 ± 9.8 % (3)	33.8 ± 2.4 %**** (4)
N-piperidinyl metonitazene	53 ± 10	1510 ± 620	3680 ± 330**
	106.9 ± 5.4 % (3)	17.0 ± 4.6 % (8)****	38.3 ± 2.8 %*** (4)
N-piperidinyl protonitazene	1.57 ± 0.16****	920 ± 280	2250 ± 400
	95.3 ± 1.8 % (3)	54.8 ± 8.9 % (3)	94.3 ± 4.2 % (4)
N-pyrrolidino etodesnitazene	27.1 ± 7.5	1740 ± 340	4900 ± 1200***
	98.1 ± 8.1 % (4)	98.3 ± 7.3 % (3)	86.9 ± 3.9 % (4)
N-pyrrolidino fluetonitazene	2.40 ± 0.25****	2490 ± 580	1580 ± 500
	110.8 ± 5.1 % (3)	69.5 ± 7.2 % (5)	111.9 ± 5.0 %*** (4)
N-pyrrolidino metodesnitazene	498 ± 17****	1860 ± 590	6400 ± 1100****
	90.6 ± 8.9 % (3)	89 ± 11 % (3)	44 ± 14 %** (4)

(n) is the number of biological replicates, each of which is conducted in duplicate.

* p < 0.05

** p < 0.01

*** p < 0.001

**** p < 0.0001.

We performed one-way ANOVA followed by Dunnett’s multiple comparison test to compare the log EC50 values of each compound to the log EC50 values of fentanyl at each receptor. % Max stimulation was calculated relative to DAMGO, U50,488H, and DPDPE for MOR, KOR, and DOR, respectively.

## Data Availability

Data will be made available on request.
